# Comparison of peak joint angles from non-concurrent squat and upper-limb assessment using a markerless Kinotek system and a marker-based Vicon system

**DOI:** 10.7717/peerj.21219

**Published:** 2026-05-13

**Authors:** Ofra A. Pottorf, Eric T. Greenberg, Micha C. Garcia, Birendra M. Dewan

**Affiliations:** 1 Department of Allied Health and Kinesiology, Hofstra University, Hempstead, New York, United States; 2 Department of Physical Therapy, New York Institute of Technology, Old Westbury, New York, United States; 3 School of Exercise and Rehabilitation Sciences, University of Toledo, Toledo, Ohio, United States; 4 Department of Physical Therapy, Long Island University, Brooklyn, New York, United States

**Keywords:** 3-D, Markerless motion capture, Kinematic analysis, Vicon, Kinotek, Biomechanics

## Abstract

**Background:**

Three-dimensional motion capture systems are essential for assessing human movement patterns, optimizing performance, and monitoring recovery. Marker-based systems are considered the gold standard but are often impractical in clinical settings due to their complexity. Markerless systems, such as Kinotek, offer a portable and user-friendly alternative. This study compared peak joint angles measured from non-concurrent trials using the Kinotek markerless system with a single-camera setup and the Vicon marker-based system.

**Methods:**

Twenty-one healthy adults performed shoulder flexion, shoulder abduction, squat, and trunk rotation movements. Movements were captured using the Kinotek markerless system and a Vicon marker-based system in separate trials in randomized immediate succession. Paired t-tests or Wilcoxon signed-rank tests compared differences in peak joint angles between the two systems, while Pearson’s (r) or Kendall’s Tau-b (τb) correlation coefficients assessed the relationship between systems. Intra-class correlation coefficients (ICC(A,5)) with 95% Confidence Intervals (CIs) evaluated within-system repeatability across five repetitions.

**Results:**

No significant differences between marker-based and markerless systems were observed for hip and shoulder flexion. Significant differences between systems were found for knee flexion, ankle dorsiflexion, trunk rotation, and shoulder abduction. Moderate correlations were found for left knee flexion (τb = 0.62) and left shoulder abduction (r = 0.58), fair correlations were found for right knee flexion (τb = 0.42), and weak or negligible correlations were found for the other assessed movements. Kinotek demonstrated lower repeatability (ICC range = −0.013–0.908) than the Vicon system (ICC range = 0.324–0.996), with good repeatability for Kinotek in ankle dorsiflexion and shoulder abduction.

**Conclusions:**

The Kinotek system demonstrated limited agreement with the Vicon system for peak joint angle measurements, with moderate or fair correlations observed in 3 of the 12 investigated variables. Significant differences between systems were identified, suggesting that the two systems should not be used interchangeably for all kinematic assessments, and highlights the need for further optimization of portable single-camera markerless technology. While the Kinotek system offers practical advantages for movement evaluation in clinical and field-based environments where traditional marker-based may be impractical, further methodological refinement and validation against gold-standard reference methods are needed before broader application in biomechanical analysis. This study adds to the growing literature on markerless motion capture by identifying areas of limited reliability alongside its practical advantages, supporting its continued development as a more accessible approach to movement analysis.

## Introduction

Motion capture technology has undergone remarkable advancements in recent decades, offering health practitioners and sport scientists valuable insights into human movement patterns. Three-dimensional (3D) motion capture systems are important for both research and clinical standpoints. The data derived from these systems can be utilized to optimize and monitor recovery progress and identify areas for potential performance enhancement (Bird et al., 2022; Daggett et al., 2022; Weinhandl et al., 2010). They are used to evaluate sports biomechanics and return to play after ligament reconstruction (Daggett et al., 2022) and assess risk for musculoskeletal injury (Hando et al., 2021). They also offer valuable clinical assessments in studying treatment effects for diverse populations including pediatric patients with hemiplegia (Mackey et al., 2005), patients post-stroke (Steffensen et al., 2023) and individuals with Parkinson’s disease (Martinez et al., 2018).

Marker-based systems are widely considered the gold standard for 3D motion analysis due to their high precision and accuracy (Brink et al., 2013; Clark et al., 2012; Dutta, 2012; Harsted et al., 2019; Pfister et al., 2014). However, their clinical utility is limited by the need for extensive setup time, large dedicated spaces, and complex post-processing requirements (Corazza et al., 2006; Dobos et al., 2022; Kanko et al., 2021; Monaghan, Delahunt & Caulfield, 2007; Perrott et al., 2017). Additionally, these systems are susceptible to several sources of error, including variability in marker placement, processing inconsistencies, and soft tissue artifacts where markers affixed to the skin shift independently from the underlying bone due to the movement of skin, muscle, and fat (Cabarkapa et al., 2022; Camomilla et al., 2018; Cappozzo et al., 1997; Corazza et al., 2006; Peters et al., 2010; Reinschmidt et al., 1997). In contrast, markerless systems use pose estimation algorithms, which avoid errors from marker-skin coupling but remain subject to landmark localization, occlusion, and noise artifacts (Keller et al., 2022; Wade et al., 2022).

While there is consensus that multi-camera markerless systems do not yet match the consistency and accuracy of marker-based systems (Scataglini et al., 2024), they demonstrate acceptable reliability and validity for spatiotemporal and kinematic measurements across various activities (Bird et al., 2022; Cabarkapa et al., 2022; Martinez et al., 2018). Despite their reduced setup complexity and elimination of physical markers, multi-camera markerless systems, like marker-based systems, still often require substantial infrastructure, including multiple synchronized cameras, a large, dedicated capture space, and post-processing requirements. Although not unique to markerless systems, some systems require repeated calibration procedures during data collection, particularly when camera views are adjusted, or the setup is altered during mid-trial. This involves recalibrating both intrinsic (*e.g*., focal length, lens distortion) and extrinsic (*e.g*., position and orientation) parameters to ensure accurate 3D reconstruction, which can be time-consuming and reduce feasibility in fast-paced or clinical environments (Noorbhai, Moon & Fukushima, 2025).

Recent technological advancements have led to the emergence of 3D motion capture through a single camera utilizing depth sensing technology, offering greater accessibility and potential for clinical integration. The portability of a single-camera setup presents a promising alternative for motion capture in rehabilitative and performance settings, enhancing the precision and objectivity of peak joint angle assessments compared to traditional methods such as goniometry and visual observation (Pottorf et al., 2023; Scott et al., 2022; Wade et al., 2022). Additionally, motion capture results are provided almost instantaneously relative to the post-processing demands of marker-based and multi-camera markerless systems (Clark et al., 2012).

Kinotek is a portable multimodal single-camera markerless system that uses the Microsoft Kinect camera for 3D motion capture. Its camera has a 3D motion-sensing device initially developed for the Xbox 360 gaming system. It utilizes a color camera combined with depth-sensing technology and internal software for skeleton-based pattern recognition (Bonnechère et al., 2014b; Otte et al., 2016). It was developed in 2010 and has undergone several subsequent technological advancements. Previous studies have compared earlier generations of the Kinect to a gold standard (Vicon Motion Systems Ltd., Oxford, UK) and found mixed results on its accuracy. For example, it demonstrated strong reliability and validity in various kinematic measurements and postural control tests such as single-leg standing balance and reaching tasks (Clark et al., 2012; Dutta, 2012), and difficulty assessing sagittal plane motions during gait such as hip, knee, and ankle flexion/extension (Bilesan et al., 2018; Eltoukhy et al., 2017; Pfister et al., 2014; Shotton et al., 2011). Limitations have also been reported for movements occurring in the transverse plane, such as trunk rotation or hip internal and external rotation (Clark et al., 2012; Rigucci, 2024). The Kinect camera has undergone improvements in its technology including higher resolution cameras and new Body Tracking Software Development Kit (SDK) (Thomas et al., 2022).

The purpose of this study was to compare peak joint angles obtained from non-concurrent repeated trials using the Kinotek markerless system and a marker-based Vicon system during selected movements in the sagittal, frontal, and transverse planes. Specifically, this study evaluated agreement, consistency, and within-system repeatability of peak joint angle measurements between systems. The primary outcome of interest was peak joint angle, a clinically relevant metric commonly used in rehabilitation and performance assessments to track changes in joint mobility, identify movement restrictions, and evaluate progress toward functional goals.

## Materials and Methods

### Ethical approval

Ethical approval was granted by the Institutional Review Board at the New York Institute of Technology Approval # BHS-1758. Participants were enrolled on a voluntary basis, and each participant provided written informed consent prior to participation in the study.

### Participants

A convenience sample of 21 healthy adults (eight female and 13 male) with a mean ± SD age of 25.1 ± 3.0 years, height of 172.9 ± 8.8 cm, and mass of 78.3 ± 12.6 kg participated voluntarily. Data from two participants encountered processing errors due to Wi-Fi connection with Kinotek and one participant encountered system processing errors with the Vicon system and were excluded from analysis. Data analysis was conducted and reported for 18 participants (*n* = 18). Participants were excluded if they were not at least 18 years old, had any allergies to adhesive, had any medical conditions that impair balance or limit safety performing the movements for this study, were taking any medications that may affect balance or ability to think clearly, had any physical pain at the time of data collection, had any tingling and/or numbness to arms, hands, legs, or feet, were not able to understand multistep instructions in the English language, or were not able to provide consent.

### Testing protocol

**Figure 1:**
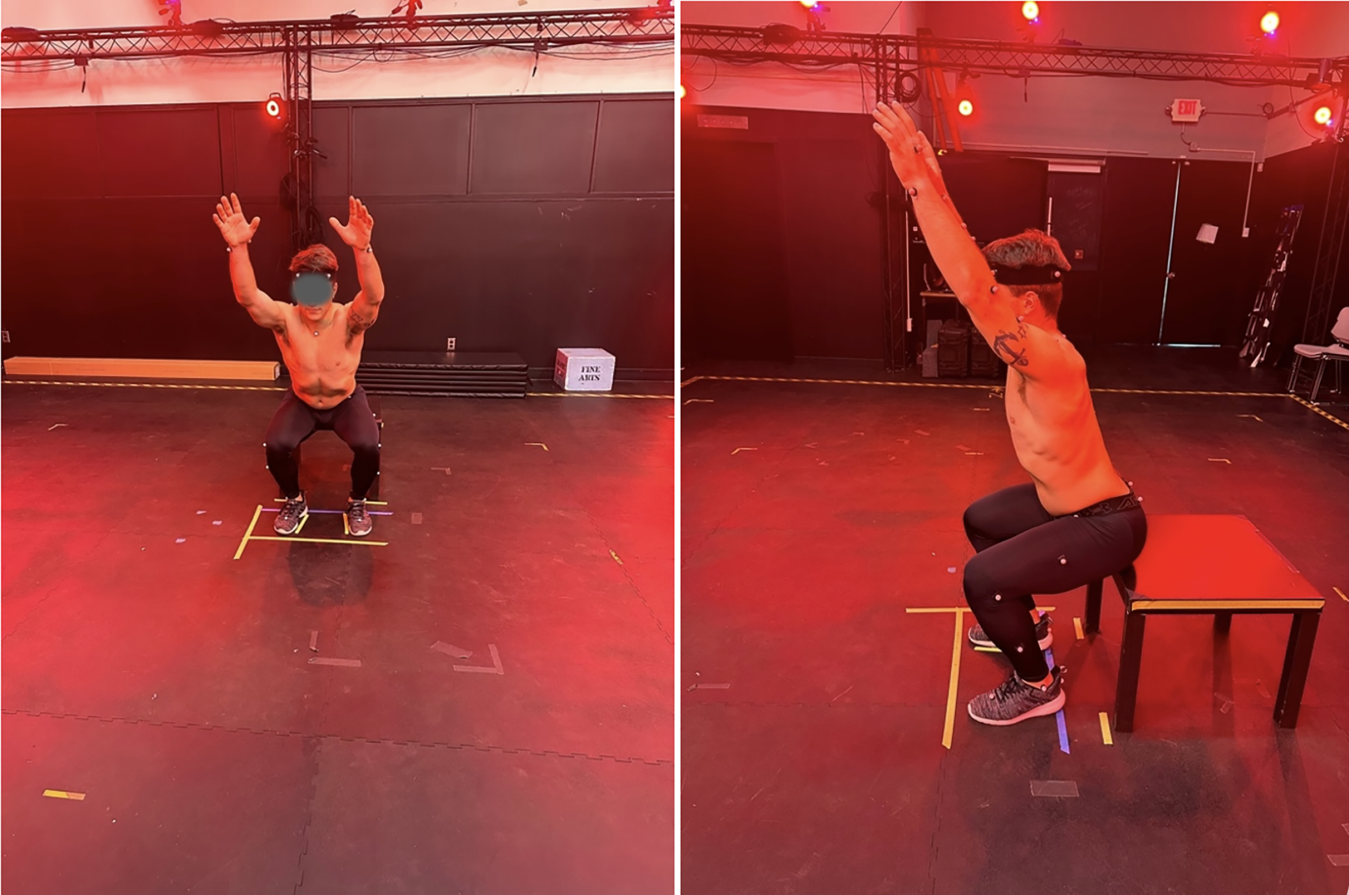
Standardization of foot positioning and squat depth across trials. Squat depth was controlled using a platform placed behind the participant, and foot placement was measured, recorded, and marked to minimize variability across testing conditions.

**Figure 2:**
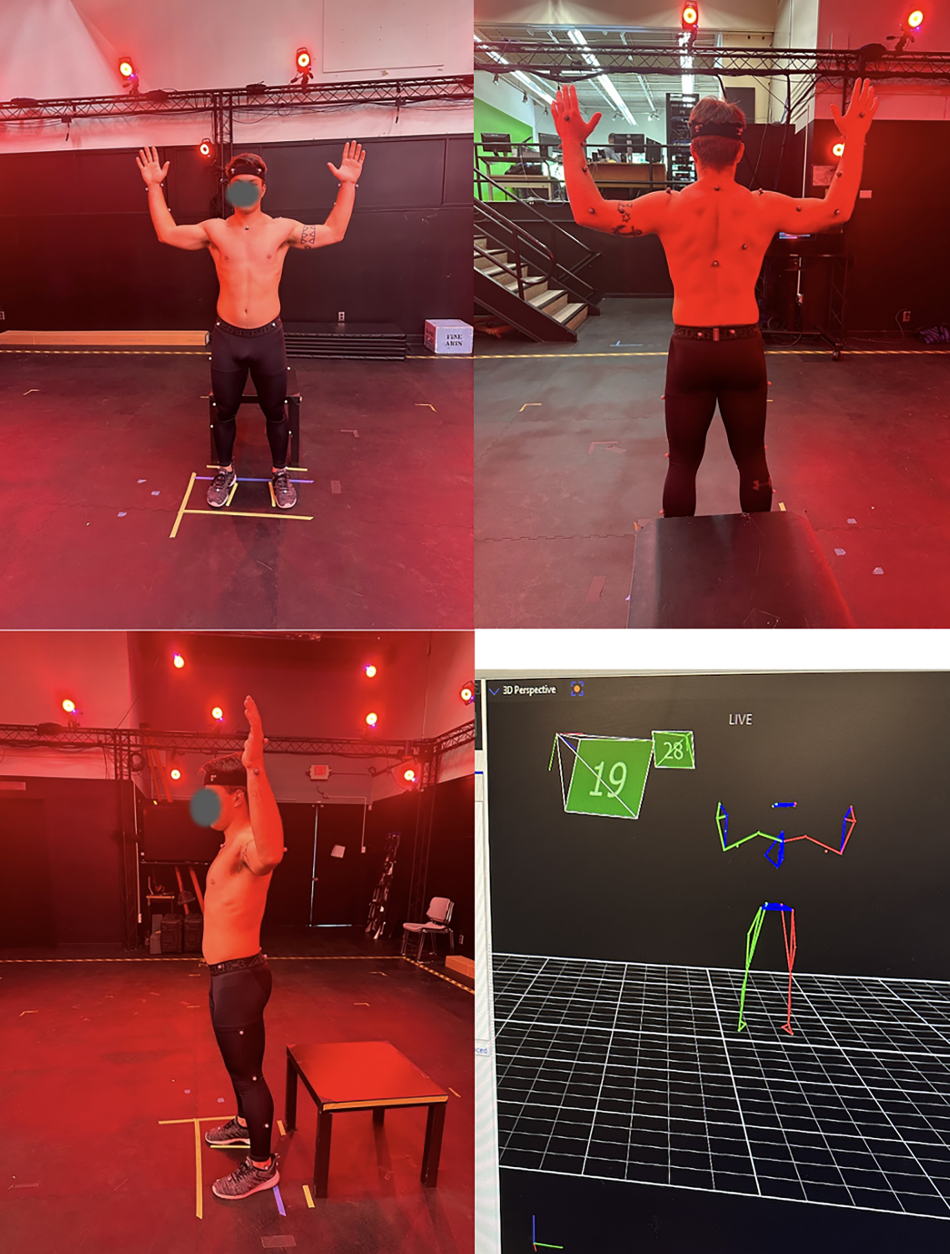
Vicon nexus full body plug-in-gait marker set model.

Following enrollment, participants attended a single visit to the New York Institute of Technology Motion Analysis Lab. Each participant performed a series of movements in standing that were analyzed by marker-based (Vicon) and markerless (Kinotek) systems. Pilot testing prior to this study indicated profound interference in the Kinotek system while operating concurrently with the Vicon system, preventing simultaneous data collection. Therefore, participants were asked to perform sequential trials of the same series of movements for marker-based and markerless systems subsequent to the other. Although the movement sequence remained consistent across participants, system order was randomized to mitigate potential order and fatigue effects, with some participants beginning with Kinotek and others with Vicon. To reduce variability in movement execution, foot placement was standardized and recorded for each participant, as illustrated in Fig. 1. Reflective markers were placed on each participant for motion capture with the Vicon system (Fig. 2) and were removed prior to data collection with the Kinotek system, ensuring that markerless motion capture was performed without reflective markers.

**Figure 3:**
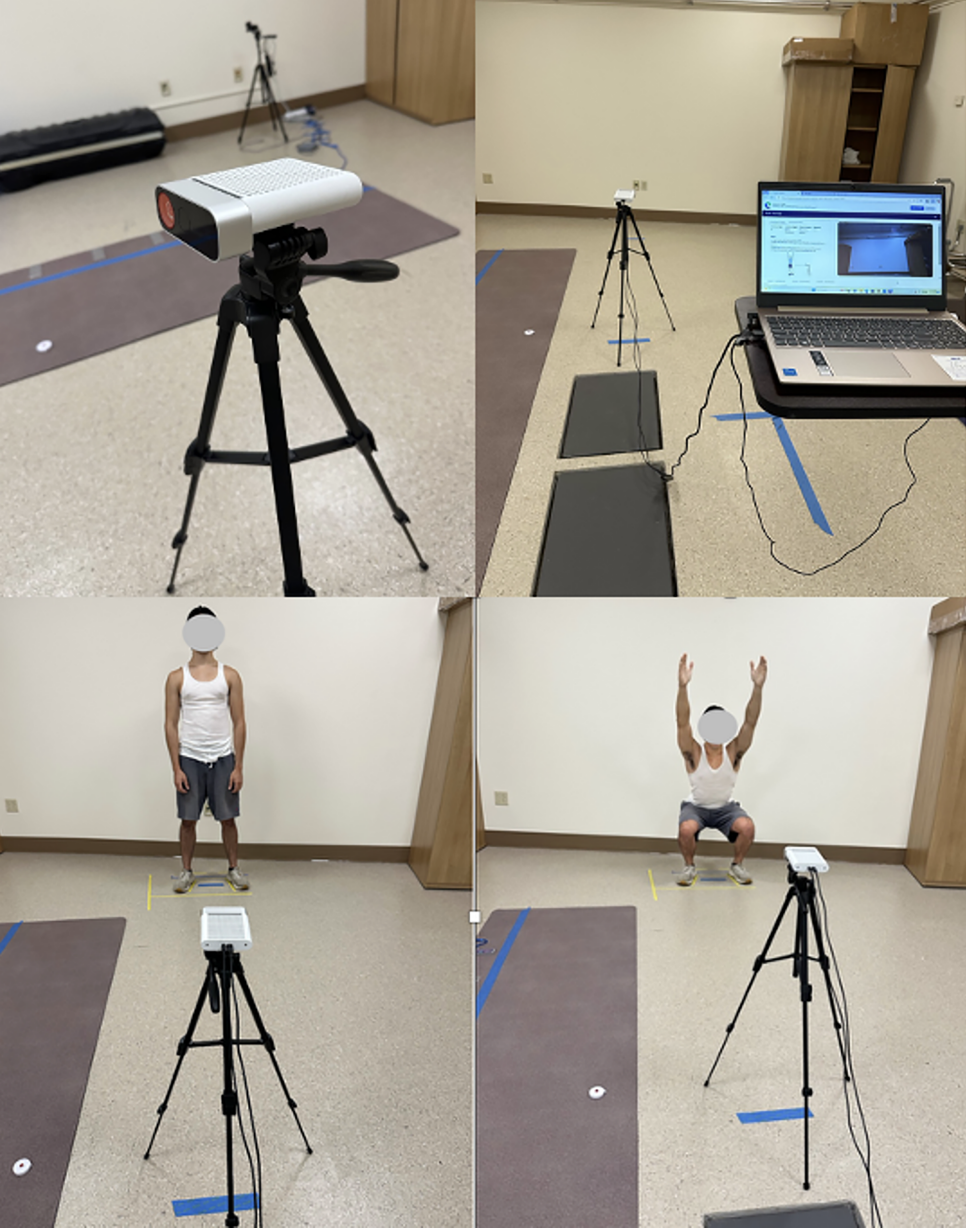
Experimental setup showing Kinotek camera on tripod connected to laptop with camera placement aligned to participant for motion capture.

The Kinotek camera was setup based on the manufacturer’s recommendations and previous methods reported (Pottorf et al., 2023); the floor was marked directly facing the camera 2.1 m from the camera’s central point. The camera was mounted on a tripod adjusted at a height aligned to the mid-pelvic region with a 6° upward tilt (Fig. 3). All movements were captured in the frontal plane; participants faced the camera standing at the 2.1 m-foot mark for movement tasks. Participants performed five repetitions of four different movements: shoulder flexion and squat (sagittal plane), shoulder abduction (frontal plane), and trunk rotation (transverse plane). These movements were selected to capture movements in the sagittal, frontal, and transverse planes while also balancing movements in the upper extremity, lower extremity, and trunk.

Movements were performed in a fixed sequence: bilateral shoulder abduction, bilateral shoulder flexion, double-leg squat, and bilateral trunk rotation. Before data collection, a physiotherapist provided uniform verbal instructions and a visual demonstration to specify arm, leg, and trunk alignment; participants practiced each task under supervised feedback. Except for squat, participants were instructed to perform each movement to the end of the available active range of motion without compensatory strategies and then completed five consecutive trials for each movement. For trunk rotation, recordings were taken with the torso rotating to the right followed by rotation to the left, avoiding cervical spine or pelvic rotation while the upper extremities remained in a fixed 90/90 position. To decrease inter-trial variability, squat depth was controlled and standardized by having the participant squat to the depth of a platform placed behind them. Foot position was measured, recorded, and marked to decrease variability across testing conditions.

### Marker-based motion capture

Joint kinematics were calculated according to the Vicon Plug-in Gait model, a marker-based biomechanical model derived from the Newington–Helen Hayes model, which defines anatomically grounded segment coordinate systems using reflective markers placed on the skin over specific anatomical landmarks and estimates joint centers through participant-specific anthropometric regression equations (Vicon Motion Systems, 2008). Joint angles were computed using the Plug-in Gait model’s predefined, joint-specific Cardan angle conventions, which vary by joint and reported variable and are applied within the Vicon software. Movements were recorded using a 26-camera Vicon Nexus optical motion capture system (Vicon Nexus version 2.7.1; Vicon Motion Systems Ltd., Oxford, UK). Three-dimensional marker trajectories were exported from Vicon Nexus, and joint angles were computed by the authors using custom MATLAB scripts. System calibration was conducted according to the manufacturer’s protocol using a Vicon Active Wand, resulting in an average global world error of less than 0.5 mm. World error was derived from image error (in pixels) and the distance from the cameras to the capture volume center. Image residuals, which represent the discrepancy between recorded and estimated marker positions on the camera sensor, were monitored throughout calibration to ensure high reconstruction accuracy. Following collection of height, weight, and anthropometric measures, participants were instrumented with 39 reflective markers according to the Vicon Nexus Full Body Plug-in Gait marker set. Three-dimensional marker trajectories were captured at 100 Hz in the mediolateral (x), anteroposterior (y), and vertical (z) directions. Woltring spline gap-filling pipelines were used to estimate marker positions during brief periods of occlusion (Woltring, 1986), and marker trajectories were filtered using a fourth-order, zero-lag, low-pass Butterworth filter with a cutoff frequency of 6 Hz to reduce high-frequency noise.

### Markerless motion capture

**Figure 4:**
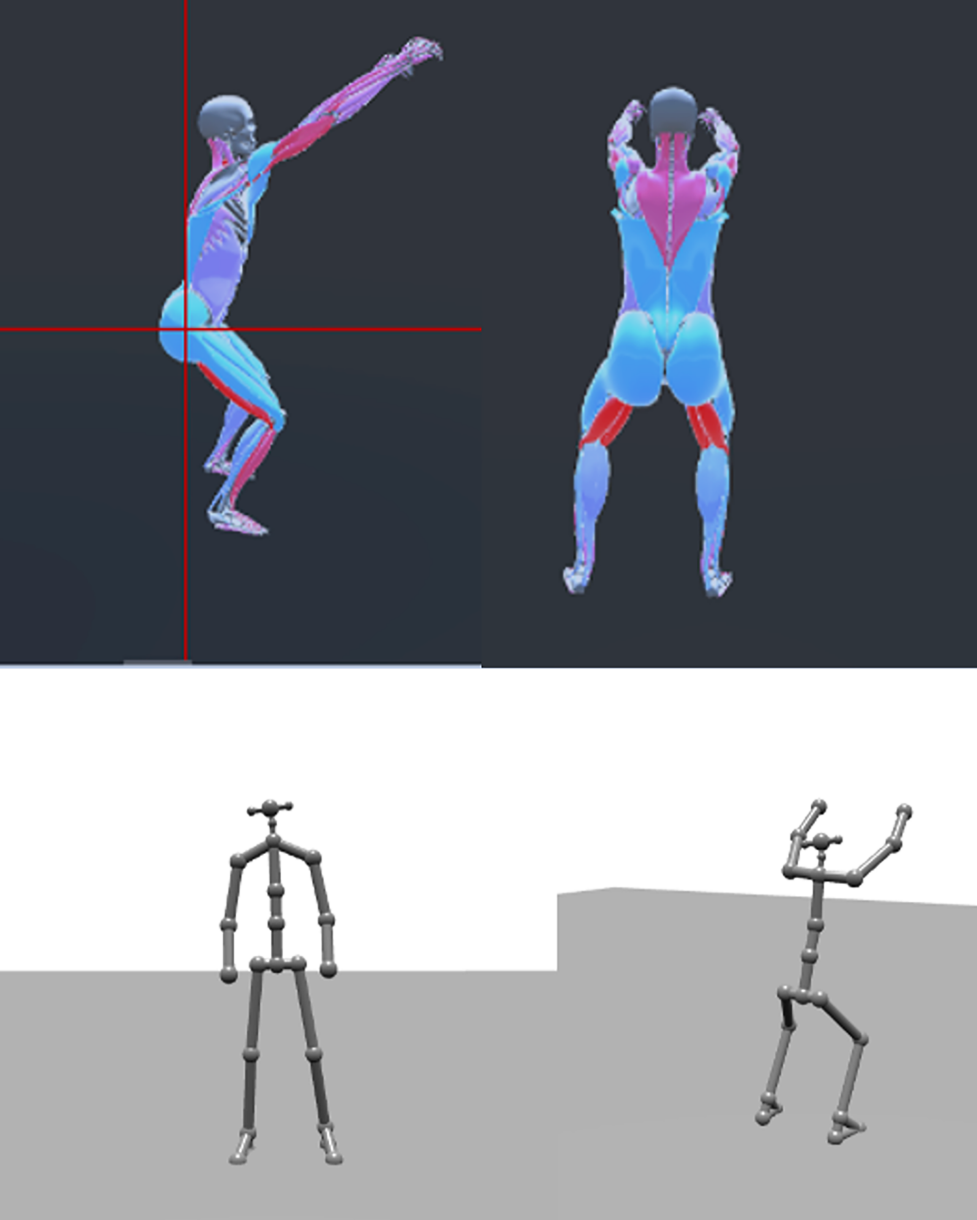
Visualization outputs from Kinotek markerless motion capture showing avatar with musculoskeletal overlay and skeletal model used for joint angle calculation during overhead squat. Plumb line added to the musculoskeletal overlay in sagittal view.

Kinotek is a multimodal markerless motion capture system that uses a depth-enhanced, single-camera setup to combine multiple sensing modalities, including Red Blue Green (RBG) video, depth Light Detection and Ranging (LiDAR) data, and machine learning-based pose estimation to generate 3D measurements of peak joint angles, movement asymmetries, and compensatory patterns. It uses the Microsoft Kinect camera. The Azure Kinect (v2) depth camera (mass = 0.44 kg; dimensions = 10.2 cm × 3.8 cm × 12.7 cm) utilizes a sampling rate of 30 frames per second. It utilizes an amplitude-modulated continuous wave time-of-flight approach to measure depth. In this method, the device emits near-infrared (NIR) light that is modulated and projected onto the environment. The sensor captures the reflected light and calculates the round-trip phase shift to estimate the distance between the camera and each point in the scene. These calculations result in the generation of a depth map, which represents the z-axis distance for each pixel, typically in millimeters. In addition to the depth map, the system also produces a “clean” infrared image that provides a grayscale representation of the scene, similar to standard infrared imaging (Microsoft, 2020). Figure 4 illustrates 3D visualizations generated from Kinotek for both a musculoskeletal overlay (avatar) and skeletal model used for joint angle calculation during an overhead squat.

Joint angles for the Kinotek system were computed internally by the proprietary Kinotek software and exported directly for analysis. Kinotek derives joint kinematics using the Azure Kinect Body Tracking SDK, which estimates 3D skeletal landmarks and segment orientations *via* depth sensing and machine learning–based pose estimation. These skeletal data are reported within a camera-centered global coordinate system, where the origin is located at the infrared camera, the X-axis extends laterally to the camera’s left, the Y-axis extends vertically (with orientation influenced by sensor tilt), and the Z-axis extends outward in the direction the camera is facing, representing depth (Albert et al., 2020). Conceptually, joint angles reflect a vector-based approach in which adjacent body segments are represented by two-point vectors defined by estimated skeletal landmarks (*e.g*., Knee→Ankle; Ankle→Foot). Rather than defining full 3D segment coordinate systems, joint angles correspond to a projected, signed in-plane angle between adjacent segment vectors, measured within a system-defined plane of motion, with the sign determined by the plane normal according to the right-hand rule. For example, thoracic rotation is defined as the signed angle between the forward-facing vectors of the Spine Chest and Pelvis joints, which represent each segment’s facing direction in the global coordinate system; this angle is calculated on a frame-by-frame basis in the XZ plane, with the sign indicating direction of rotation. All joint angle computations are performed entirely within the Kinotek software environment and are not user-modifiable.

For each recording session, the system automatically selects the peak rotation value, the highest signed angle recorded during the movement, as the representative value for analysis. The plane of motion, plane normal used to assign sign, and any projection of segment vectors into that plane are defined internally by the software and are not user-configurable. Accordingly, joint angles were not recomputed by the authors in participant-specific anatomical coordinate systems or movement-derived frames, and no additional joint angle calculations were performed for the Kinotek data. The joint angle definitions provided in this study therefore describe the operational interpretation of the reported markerless outputs rather than an explicit biomechanical reconstruction of joint kinematics. Although camera-based planes of motion may not align with anatomical planes, particularly for multi–degree-of-freedom joints, this approach reflects how single-camera markerless motion capture systems are typically used in applied clinical and field settings, where users rely on system-generated joint angle outputs.

### Statistical analysis

Joint kinematics and marker trajectories were imported into a custom MATLAB program (The MathWorks, Natick, MA, USA). Bilateral peak joint angles in five trials were averaged for each movement for comparison. Shapiro-Wilk tests were used to assess the normality of data sets. Comparison of peak joint angles between Kinotek and the marker-based system were made using paired t-test or Wilcoxon signed-rank test if not normally distributed. The correlation between the measurements from two systems was examined with a Pearson’s product moment correlation coefficient (r) or Kendall’s Tau-b correlation coefficient (τb) if not normally distributed. Strength of correlations were based on the scales described by Akoglu (2018), where values of 0.90 or higher indicate a very strong correlation, 0.70–0.89 strong, 0.50–0.69 moderate, 0.30–0.49 fair, and 0.10–0.29 poor, with values below 0.10 considered negligible. The Mean Difference (MD) was reported with a 95% Confidence Interval (CI). The 95% CI for the mean differences were calculated using the paired t-distribution (mean difference ± t(df, 0.975) × standard error of the paired differences).

Statistical analyses were performed using IBM SPSS Statistics for Windows, version 27 (IBM Corp., Armonk, NY, USA). The significance level was set at α = 0.05 (two-tailed). Bland-Altman analysis was performed to assess the level of agreement between systems. Differences between systems were calculated as Kinotek–Vicon; thus, a positive bias indicates higher values from Kinotek. The limits of agreement were calculated as the mean bias ± 1.96 × SD of the differences. Within-session repeatability of each motion capture system was assessed separately for Kinotek and Vicon. For each participant, five repeated trials were collected within a single session and entered directly into SPSS. Consistent with recommendations by McGraw & Wong (1996), intraclass correlation coefficients (ICC(A,5)) were calculated using a two-way mixed-effects model with absolute agreement, considering the average of five measurements (average measures), to quantify within-session repeatability (precision) of each system. ICC values were interpreted as poor (<0.50), moderate (0.50–0.75), good (0.75–0.90), or excellent (>0.90), in accordance with Koo & Li (2016).

## Results

**Figure 5:**
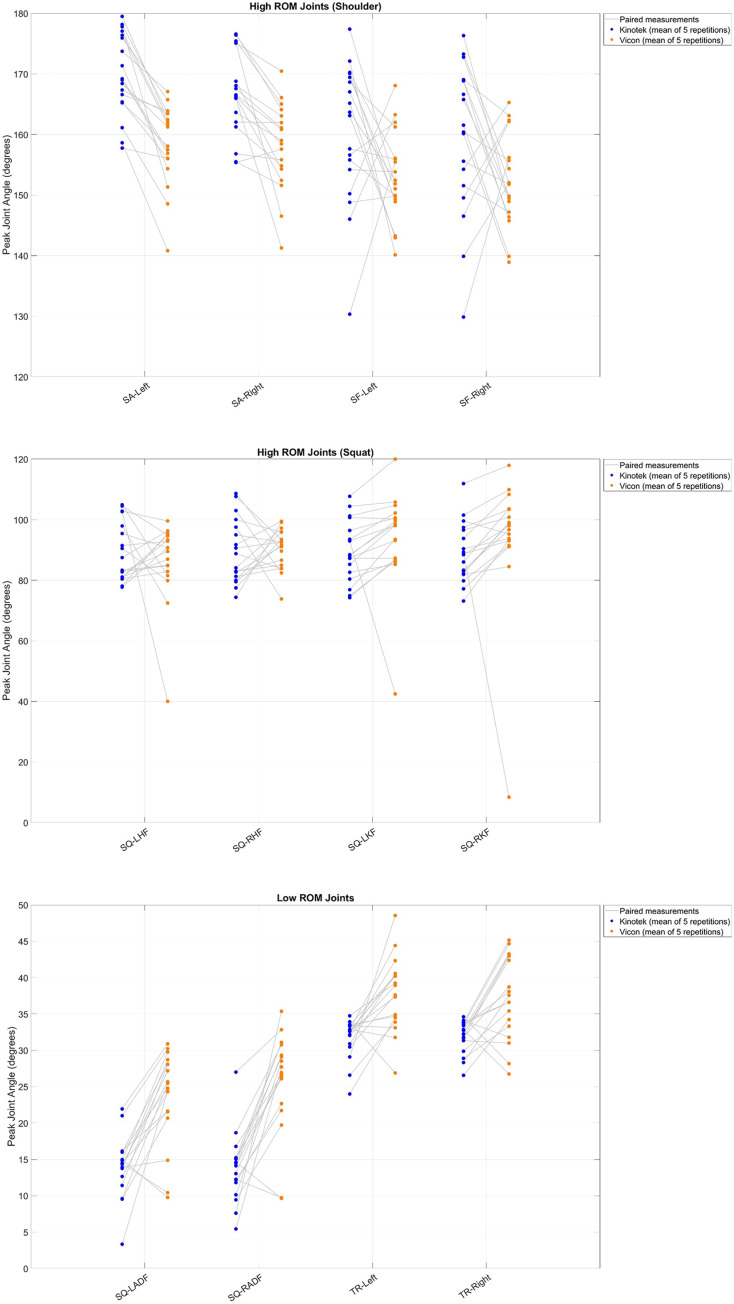
Measurement differences in peak joint angles across low- and high-range of motion movements between Kinotek and Vicon. Each data point represents the mean of five repetitions (A) presents High-ROM joints (Shoulder), (B) presents High-ROM joints (Squat), and (C) presents Low-ROM joints. Paired data points represent individual participant measurements from both systems. SA_Left, Shoulder Abduction-Left; SA_Right, Shoulder Abduction-Right; SF_Left, Shoulder Flexion-Left; SF_Right, Shoulder Flexion-Right; SQ_LHF, Squat-Left Hip Flexion; SQ_RHF, Squat-Right Hip Flexion; SQ_LKF, Squat-Left Knee Flexion; SQ_RKF, Squat-Right Knee Flexion; SQ_LADF, Squat-Left Ankle Dorsiflexion; SQ_RADF, Squat-Right Ankle Dorsiflexion; TR_Left, Trunk Rotation-Left; TR_Right, Trunk Rotation-Right.

**Figure 6:**
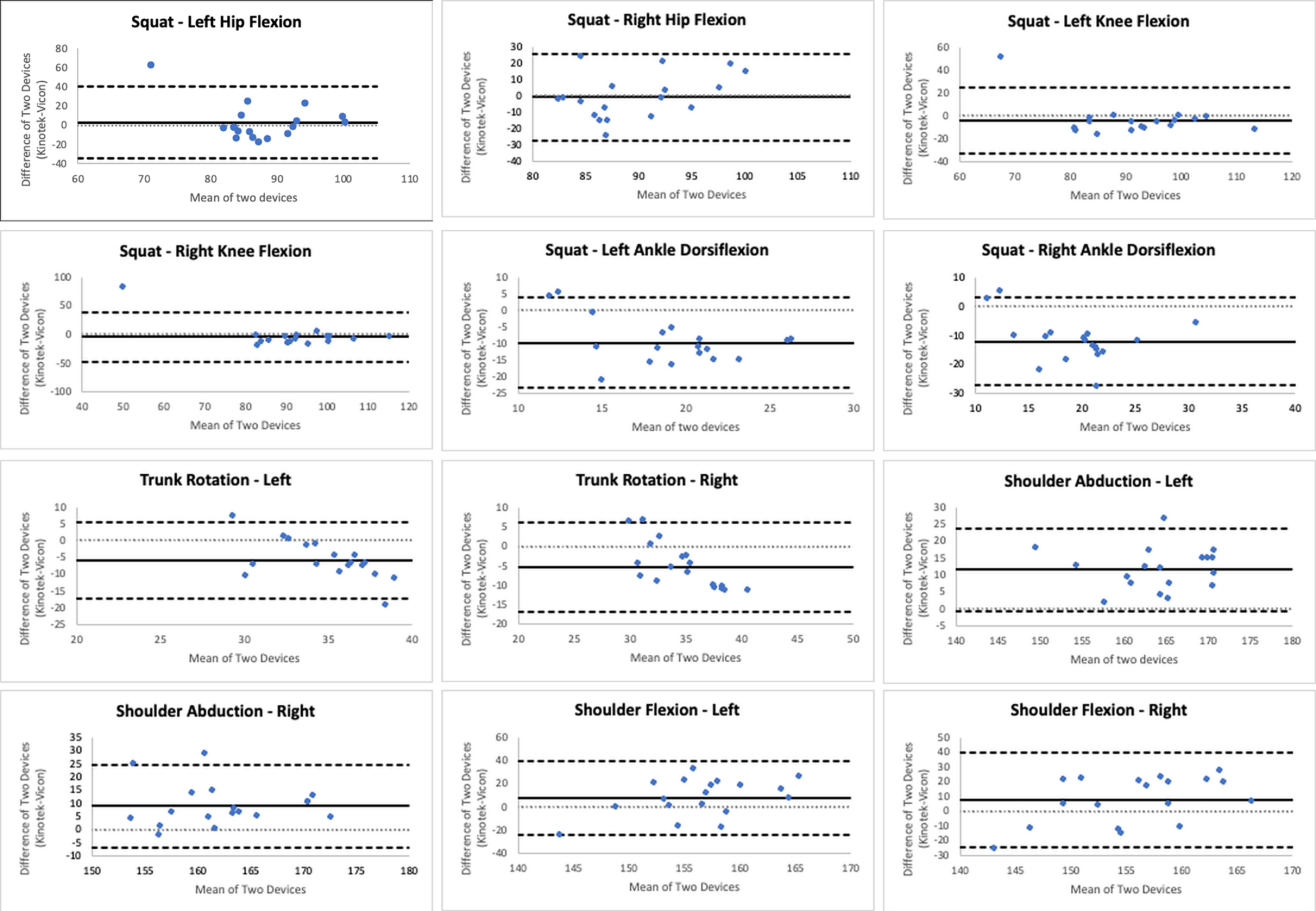
Bland-altman plots with limits of agreement (mean difference ± 1.96 × SD) for the difference between Kinotek and Vicon. Solid black horizontal line indicates mean difference, dashed black horizontal lines indicate upper and lower limits of agreement.

Figures 5A–5C illustrate plots of individual participant data for each joint and side and connect the paired measurements (Kinotek and Vicon) with a single line segment, while also grouping joints into low and high-range of motion. The mean (SD) of peak joint angles for both systems, along with the mean difference (95% CI), statistical test of difference, and correlation between the two systems, are reported in Table 1. These analyses together describe the consistency (correlation), agreement (bias and limits of agreement), and within-system repeatability between systems. There were no statistically significant differences in the peak joint angles for bilateral hip flexion and shoulder flexion between Kinotek and the marker-based system (*p* > 0.05). Significant differences (*p* < 0.05) were detected in peak joint angles for bilateral knee flexion, ankle dorsiflexion, trunk rotation, and shoulder abduction. Outlier inspection revealed two outliers for knee flexion (left and right) originating from the same participant, and one outlier for left hip flexion from another participant. Removing the outliers did not change the statistical significance of the results; hence, the data were retained. Bland-Altman plots are reported in Fig. 6. In the Bland-Altman plots, the mean biases were small for most joint angles, indicating minimal systematic differences between the two systems. However, a downward trend was observed for trunk rotation angles with Kinotek underestimating participants with greater peak joint angles. Reliability was evaluated using ICC(A,5) with CI of 95% (Table 2). Interpretation focused not only on point estimates but also on the precision of these estimates, with wider CIs reflecting greater uncertainty in within-system repeatability despite statistically significant point estimates. Kinotek demonstrated poor reliability for bilateral hip flexion, knee flexion, trunk rotation, and shoulder flexion. Reliability was moderate for left ankle dorsiflexion, good for right ankle dorsiflexion and right shoulder abduction, and excellent for left shoulder abduction. In contrast, Vicon exhibited generally higher reliability. Poor reliability was observed only for bilateral hip flexion. Reliability was moderate for left knee flexion, good for left ankle dorsiflexion and bilateral shoulder abduction, and excellent for right knee flexion, right ankle dorsiflexion, bilateral trunk rotation, and bilateral shoulder flexion. All reliability coefficients were statistically significant (*p* < 0.05), with the exception of Kinotek right trunk rotation.

**Table 1:** Peak joint angles during different movements.

Movement		N	Kinotek Mean° (SD)	Vicon Mean° (SD)	Mean Diff.° (95% CI)	*p*-value Paired Diff.	Correlation	
							Coeff.	*p*-value
Squat	Left Hip Flexion^a^	18	89.1 (9.8)	86.1 (13.4)	3.0 [−6.5 to 12.5]	0.913^b^	Τb = −0.16	0.363
	Right Hip Flexion	18	89.2 (10.3)	90.3 (6.9)	−1.1 [−7.9 to 5.7]	0.739	r = −0.24	0.341
	Left Knee Flexion^a^	18	89.8 (9.7)	93.9 (15.4)	−4.1 [−11.3 to 3.1]	**0.004** ^b^	Τb = 0.62	**<0.001**
	Right Knee Flexion^a^	18	89.4 (10.0)	94.0 (22.6)	−4.6 [−15.7 to 6.5]	**0.006** ^b^	Τb = 0.42	**0.015**
	Left Ankle Dorsiflexion^a^	18	14.2 (4.0)	23.9 (6.5)	−9.7 [−13.1 to −6.3]	**<0.001**	Τb = 0.26	0.129
	Right Ankle Dorsiflexion^a^	18	13.5 (4.9)	25.6 (7.0)	−12.1 [−15.9 to −8.3]	**<0.001**	Τb = 0.08	0.649
Trunk Rotation	Thoracic Spine Left^a^	18	31.9 (2.6)	37.9 (5.2)	−6.0 [−8.9 to v3.1]	**<0.001**	Τb = 0.04	0.820
	Thoracic Spine Right^a^	18	32.1 (2.2)	37.4 (5.7)	−5.2 [−8.2 to −2.3]	**0.004** ^b^	Τb = 0.03	0.879
Shoulder Abduction	Left	18	170.0 (6.9)	158.4 (6.7)	11.5 [8.5 to 14.6]	**<0.001**	r = 0.58	**0.012**
	Right	18	166.8 (7.0)	158.0 (7.1)	8.8 [4.8 to 12.7]	**<0.001** ^b^	r = 0.37	0.133
Shoulder Flexion	Left	18	160.2 (11.5)	152.9 (7.4)	7.3 [−0.7 to 15.3]	0.072	r = −0.43	0.076
	Right	18	159.5 (12.6)	152.3 (7.5)	7.2 [−0.9 to 15.3]	0.064^b^	r = −0.28	0.260

**Notes:**

N, Sample size, SD, Standard deviation; CI, Confidence Interval; Diff., Difference; Coeff., Coefficient; r = Pearson’s product moment correlation coefficient. Bold = Statistically significant values

a Not normally distributed–Kendall tau-b correlation coefficient (τb).

b Paired difference not normally distributed–Wilcoxon Signed-rank test.

**Table 2:** Within-session repeatability (ICC, 95% CI) of Kinotek and Vicon for peak joint angles, calculated separately for each system using a two-way mixed-effects model with absolute agreement, based on the average of five repeated trials.

Movement		N	Kinotek ICC (95% CI)	*p*-value	Vicon ICC (95% CI)	*p*-value
Squat	Left hip flexion	18	0.329 [0.131–0.588]	<0.001	0.474 [0.266–0.705]	<0.001
	Right hip flexion	18	0.356 [0.154–0.612]	<0.001	0.324 [0.126–0.584]	<0.001
	Left knee flexion	18	0.414 [0.208–0.659]	<0.001	0.653 [0.462–0.825]	<0.001
	Right knee flexion	18	0.420 [0.214–0.663]	<0.001	0.996 [0.992–0.998]	<0.001
	Left ankle dorsiflexion	18	0.701 [0.522–0.853]	<0.001	0.877 [0.780–0.945]	<0.001
	Right ankle dorsiflexion	18	0.764 [0.607–0.888]	<0.001	0.952 [0.909–0.979]	<0.001
Trunk rotation	Left	18	0.138 [−0.015 to 0.392]	=0.043	0.914 [0.841–0.962]	<0.001
	**Right**	**18**	−0.013 [−0.108 to 0.181]	**=0.542**	0.928 [0.866–0.969]	<0.001
Shoulder abduction	Left	18	0.908 [0.831–0.959]	<0.001	0.875 [0.775–0.944]	<0.001
	Right	18	0.830 [0.704–0.922]	<0.001	0.859 [0.751–0.936]	<0.001
Shoulder flexion	Left	18	0.176 [0.010–0.437]	=0.005	0.923 [0.857–0.966]	<0.001
	Right	18	0.226 [0.047–0.491]	=0.018	0.934 [0.876–0.971]	<0.001

**Note:**

ICC, Intraclass correlation coefficient; CI, Confidence interval; N, Sample size; Bold = Non-significant values.

## Discussion

The purpose of this study was to compare peak joint angles obtained from non-concurrent trials using the Kinotek markerless motion capture system and a gold-standard marker-based Vicon system across selected movements and planes of motion, with particular emphasis on agreement, consistency, and within-system repeatability. It is important to note that a high correlation does not necessarily imply close agreement, as strong consistency in measurement trends can coexist with systematic bias. The findings suggest that peak joint angle measurements showed moderate correlations between the systems for left knee flexion (τb = 0.62) and left shoulder abduction (r = 0.58), and a fair correlation for right knee flexion (τb = 0.42). The correlations for other joint angles were not statistically significant (*p* > 0.05). Although some statistically significant differences were observed, they may reflect inherent variations in the systems’ algorithmic processing and underlying biomechanical models. Overall, findings from this study are consistent with emerging literature demonstrating that markerless motion capture technologies have the potential to produce similar movement trends for select tasks, although notable differences between systems remain. Limitations in markerless technologies still exist that require further advancements to improve agreement, consistency, and within-system repeatability across joints of the body.

Findings from the present study support previous literature demonstrating similar kinematic data comparability between multi-camera markerless and marker-based systems (Clark et al., 2012; Harsted et al., 2019; Dobos et al., 2022; Mauntel et al., 2021; Otte et al., 2016; Park et al., 2022; Perrott et al., 2017; Van Hooren et al., 2023). For instance, Dutta (2012) compared the Kinect to a Vicon system for a variety of movements occurring in the (x), (y), and (z) axes during workplace ergonomic assessments and reported root-mean-squared (RMS) position errors, representing the average 3D distance between corresponding marker coordinates, of 0.0065 m (x-axis), 0.0109 m (y-axis), and 0.0057 m (z-axis). Although these values reflect positional rather than angular accuracy, they are relevant to joint-angle comparisons because angular kinematics are derived from the relative positions of body segments. Given that a 1 cm positional deviation can correspond to several degrees of angular difference depending on segment length and rotation axis, the 3–12° joint-angle differences observed in our study are consistent with the magnitude of positional error reported by Dutta (2012). Thus, while the Kinect and similar systems exhibit slightly lower spatial accuracy than marker-based systems, their measurement fidelity remains within acceptable limits for estimating peak joint angles. In a study conducted by Eltoukhy et al. (2017), the authors compared the reliability of the Kinect to marker-based systems and concluded that the Kinect system is an acceptable tool for assessing rotations in the x-axis within the sagittal plane, including hip and knee flexion joint angles throughout the gait cycle. However, similar to our study, they acknowledged limitations in the Kinect’s ability to capture ankle joint kinematics, reporting significant underestimations compared to marker-based motion capture systems. Naeemabadi et al. (2019) further identified that the Azure Kinect exhibited increased noise interference, particularly when joints had a relatively small joint angle or when bone segments were shorter, which may explain the discrepancies observed between systems in the measurement of ankle dorsiflexion and trunk rotation.

In the present study, no statistically significant differences in peak joint angles were found for bilateral hip flexion, which contrasts with previous reports of inaccuracies in measuring hip flexion with the Kinect during gait (Mentiplay et al., 2015; Pfister et al., 2014). However, consistent with prior findings, larger discrepancies were observed when measuring ankle dorsiflexion. Jamali & Behzadipour (2016) and Kharazi et al. (2015), similarly reported errors in tracking ankle joint angles using the Kinect, which may be due to focal length or lens distortion in its sensor technology when tracking the foot relative to the lower leg. In addition, Kinotek defines the shank as the Knee→Ankle vector and the foot as the Ankle→Foot vector. Because the foot is represented by a single distal landmark (Foot), small tracking errors can disproportionately affect foot orientation, which may partly explain why Kinotek underestimated ankle motion relative to Vicon.

The present study identified negligible correlations between the Azure Kinect and a marker-based motion capture system for bilateral trunk rotation, suggesting limitations in the Kinect’s ability to accurately capture transverse plane movements. Because Kinotek and Vicon utilize different kinematic models and joint definitions, direct comparisons of peak joint angle values may reflect systematic differences between systems. However, each system applies its definitions consistently, allowing for valid assessment of correlations and relative agreement. These findings are consistent with previously reported limitations of earlier Kinect versions. Clark et al. (2012) noted that the Microsoft Kinect (v1) lacked the capability to accurately assess internal and external joint rotations due to its inability to define an orthogonal axis not directly related to a joint center. This technical constraint affects the Kinect’s ability to resolve rotational components that occur around the longitudinal axis of a segment, such as trunk rotation, because it cannot generate a reliable coordinate frame orthogonal to the primary axis of motion. As a result, angular data for axial or transverse plane movements may be misrepresented or underestimated, particularly when the motion does not involve large displacements or easily detectable changes in orientation.

While the Azure Kinect represents a refinement of the original technology, it may still exhibit inherent limitations in capturing transverse plane kinematics, which could partly explain the observed discrepancies in trunk rotation measures in the present study. Prior validation studies of the Azure Kinect have similarly reported large errors during movements occurring in the transverse plane (Clark et al., 2012; Rigucci, 2024), and although trunk mobility has been examined using the Azure Kinect, these investigations have primarily focused on motion in the sagittal and frontal planes, with limited evidence addressing its validity for capturing trunk rotation in the transverse plane (Osmanoğlu et al., 2025; Foreman & Engsberg, 2020).

The ICC analysis showed clear differences in within-session repeatability between the two motion capture systems. Vicon demonstrated consistently good to excellent repeatability across most joints and movements, whereas Kinotek showed greater variability, with repeatability strongly dependent on joint and task. Kinotek demonstrated lower repeatability for trunk rotation and proximal joint kinematics, while higher repeatability was observed for ankle dorsiflexion and shoulder abduction. These findings suggest that within-session precision of markerless motion capture may be more sensitive to movement complexity and joint-specific estimation challenges, whereas marker-based systems provide more stable measurements across a wider range of joint motions.

Three outliers were identified in the motion capture data. Two of these values occurred in left and right knee flexion and originated from the same participant, while one outlier was observed for left hip flexion in a different participant. Inspection of the paired measurements indicated that these extreme values were associated with the Vicon marker-based system. Although marker-based motion capture systems are widely considered the gold standard, outliers can still occur due to several well-documented sources of error. One potential explanation is soft tissue artifact, in which markers attached to the skin move relative to the underlying bone during dynamic movements, leading to inaccuracies in joint angle estimation (Camomilla et al., 2018; Peters et al., 2010; Reinschmidt et al., 1997). In addition, marker placement variability or minor deviations in anatomical landmark identification can influence the definition of segment coordinate systems and joint centers used in biomechanical modeling (Cappozzo et al., 1997). Temporary marker occlusion, mislabeling, or trajectory reconstruction artifacts during data processing may also produce anomalous kinematic values in optical motion capture systems (Pfister et al., 2014). Notably, removal of these observations did not alter the statistical significance of the comparisons; hence, all data were retained for analysis. While the outliers identified in this study were associated with the marker-based system, it is important to note that both marker-based and markerless motion capture technologies are susceptible to measurement errors under certain conditions.

For markerless systems such as Kinotek, potential sources of error are often related to the time-of-flight technology used by the Microsoft Azure Kinect. Because this method measures depth based on reflected infrared light, accuracy can be reduced when tracking dark surfaces that absorb light or when body parts become occluded from the camera’s view during dynamic movements, preventing the sensor from capturing accurate depth data and leading to occasional tracking errors or outlier values (Noorbhai, Moon & Fukushima, 2025). Technologies such as Kinotek rely on RGB and depth sensors to estimate joint positions, making them susceptible to occlusion, sensor misalignment, and environmental interference (Mentiplay et al., 2015; Pfister et al., 2014). Occlusion, where body segments obscure one another, can lead to tracking errors, particularly during dynamic movements such as squats, jumps, or directional changes (Stone et al., 2013). Additionally, camera angle and placement can influence data integrity; non-optimal perspectives may distort joint estimation or lead to dropped frames (Bonnechère et al., 2014a, 2014b; Clark et al., 2013). Environmental factors including lighting conditions, color contrast, and body morphology may also affect skeletal tracking by degrading depth perception and joint contour accuracy (Noorbhai, Moon & Fukushima, 2025). Furthermore, the algorithms used in markerless systems may introduce additional variability through smoothing or filtering artifacts or incorrect joint labeling when movements deviate from trained biomechanical models (Giblin et al., 2016). Previous literature comparing Kinect-based systems to gold-standard marker-based technologies, such as Vicon, has consistently reported larger measurement errors, particularly in lower limb kinematics during tasks like gait or jump landings (Mentiplay et al., 2015; Pfister et al., 2014). To mitigate these issues in future work, researchers have recommended multi-angle sensor configurations, strict calibration protocols, and targeted post-processing techniques to improve the accuracy and reliability of markerless motion capture (Clark et al., 2013; Stone et al., 2013). Collectively, these factors highlight that both marker-based and markerless motion capture systems may occasionally produce anomalous values, particularly during dynamic multi-joint movements.

This study had several limitations. First, the participant cohort consisted of a convenience sample of healthy individuals, which limits the generalizability of the findings to broader or clinical populations. Second, the study focused exclusively on peak joint angles, which does not capture the full complexity of movement patterns or intersegmental coordination. Additionally, the Vicon Plug-in Gait model computes 3D joint angles using Cardan angle decomposition based on anatomically defined segment coordinate systems derived from marker trajectories. This method, derived from the Newington–Helen Hayes gait model, has been validated in both clinical and research settings. Differences in calculated joint angles between the systems may be attributed to variations in underlying technology and biomechanical modeling approaches. The aim of the study was therefore to compare the two systems and examine the level of agreement, consistency, and within-system repeatability for kinematic measurements produced by Kinotek relative to those from the Vicon system. Kinotek provides a rapid and user-friendly setup with immediate access to kinematic data, making it a potentially valuable tool for clinical practice. Therefore, it is essential to assess whether its level of agreement is sufficient to support clinical decision-making.

Another important limitation is that data were collected sequentially rather than simultaneously. Concurrent data collection using the Azure Kinect and Vicon motion capture systems was not feasible due to spectral and temporal overlap in the IR range. The Azure Kinect utilizes time-of-flight infrared sensing at an approximate wavelength of 850 nm, which overlaps with the strobing signals emitted by Vicon’s optical systems (MX3 and T40 cameras wavelength ~780 nm). Operating both systems simultaneously within this spectral band resulted in IR interference, leading to compromised depth quality and tracking accuracy from the Kinect camera. This issue has been previously documented by Naeemabadi et al. (2019), who showed that retroreflective markers can reflect IR light and introduce noise into the Kinect’s depth sensor, thus reducing the accuracy of kinematic outputs. This phenomenon was also reported by Naeemabadi et al. (2018), who found interference with the Azure Kinect when operating it concurrently with a marker-based system. While some studies have reported simultaneous data collection, this approach is contingent upon synchronization of Vicon’s strobe intensity and timing relative to the markerless system. For example, Thomas et al. (2022) reported concurrent use of Azure Kinect and a 12-camera Vicon system during a sit-to-stand task, although the specific IR wavelengths used were not reported and the authors acknowledge that systematic differences are possible due to marker placement. Naeemabadi et al. (2019) further noted that although simultaneous use of marker-based and markerless systems may be possible when IR wavelengths differ, full mitigation of interference has yet to be resolved. Proposed strategies include minimizing the use of reflective markers and repositioning them away from critical tracking zones, such as joint axes. However, a standardized protocol for ensuring interference-free and accurate simultaneous data capture using Azure Kinect and optical motion capture systems has yet to be established. To address this limitation, participants were instructed to perform the same sequence of movements for both the Kinotek and the marker-based system in immediate succession order along with an implemented standardized protocol to limit variability.

An important consideration is that Kinotek and Vicon rely on fundamentally different kinematic models and computational approaches. Kinotek joint angles are generated internally using a proprietary, vector-based orientation method derived from estimated skeletal landmarks, whereas Vicon joint angles are computed using an anatomically defined model with Cardan angle decomposition. As a result, differences in peak joint angle values may reflect both measurement error and inherent differences in kinematic definitions rather than true biomechanical disagreement.

Findings from the present study align with prior research evaluating the performance of markerless motion capture systems. Eltoukhy et al. (2017) reported that while 3D markerless technologies demonstrate considerable potential, inconsistencies in their reliability and validity persist, emphasizing the ongoing need for methodological refinement. These systems generally provide good to excellent agreement in capturing spatiotemporal and kinematic variables, particularly when used under controlled conditions. More recent work by Jia et al. (2025) supports this conclusion, showing that the accuracy of the Azure Kinect is influenced by experimental factors such as participant-to-sensor distance, body orientation, self-occlusion, and lighting. They also found improved performance in static conditions compared to dynamic tasks, suggesting current limitations in real-time movement tracking. Despite these challenges, there is a growing body of evidence supporting the continued integration of systems like Kinotek into motion analysis workflows, particularly in settings where rapid, marker-free assessments are desired (Antico et al., 2021; Jia et al., 2025).

Despite advancements in markerless motion capture technology, these systems still encounter significant challenges when compared to the gold standard of marker-based systems, including issues such as occlusion, noise, and computational complexity. Currently, the evidence is inconclusive on whether markerless systems are accurate, valid, or reliable for measuring kinematic variables (Cimolin & Galli, 2014; Colyer et al., 2018). However, our findings indicate that some peak joint angle measurements obtained from the Kinotek system are comparable to those from multi-camera markerless systems. Nonetheless, marker-based systems are still considered the gold standard for 3D motion analysis. To further advance markerless technologies, it is essential to address ongoing challenges such as technical limitations and measurement discrepancies caused by technology-specific interferences (Perrott et al., 2017).

## Conclusions

The Kinotek markerless motion capture system demonstrated limited agreement with the marker-based Vicon system for peak joint angle measurements, with significant differences observed for several variables, including knee flexion, ankle dorsiflexion, trunk rotation, and shoulder abduction, while no significant differences were found for hip flexion and shoulder flexion. Consistency between systems was limited, with moderate or fair correlations observed in only 3 of the 12 variables examined. Within-system repeatability also differed substantially between systems, with Kinotek demonstrating greater variability and generally lower ICC values compared to the Vicon system. While the Kinotek system offers advantages in portability, rapid setup, and accessibility, further refinement and validation are required before it can be considered a viable alternative to marker-based motion capture in biomechanical research. The simplicity of a single-camera 3D markerless system offers a practical solution for clinical and field assessments, however, traditional marker-based systems remain the gold standard for reliability and validity in research-based motion analysis. Future research should focus on improving markerless motion capture technologies by addressing known limitations, optimizing these systems for broader applications, and exploring their utility in clinical populations and rehabilitation settings.

## Supplemental Information

10.7717/peerj.21219/supp-1 Supplemental Information 1 Raw Data.

## Additional Information and Declarations

### Data Availability

The following information was supplied regarding data availability:

The raw data are available in the Supplemental Files.
